# Correction to: Biomedical evidence engineering for data-driven discovery

**DOI:** 10.1093/bioinformatics/btad026

**Published:** 2023-01-27

This is a correction to: Sendong Zhao, Aobo Wang, Bing Qin, Fei Wang, Biomedical evidence engineering for data-driven discovery, *Bioinformatics*, Volume 38, Issue 23, 1 December 2022, Pages 5270–5278, https://doi.org/10.1093/bioinformatics/btac675

In the originally published version of this manuscript, affiliations 1 and 2 were incorrect and given as follows:

1 Department of Population Health Sciences, College of Computer Science and Technology, Harbin Institute of Technology, Harbin 10065, China,

2 Department of Population Health Sciences, College of Science, Australian National University, Canberra, ACT 2600, Australia

Both affiliations have now been corrected online.

